# On exploring and ranking risk factors of child malnutrition in Bangladesh using multiple classification analysis

**DOI:** 10.1186/s40795-017-0194-7

**Published:** 2017-09-07

**Authors:** Kakoli Rani Bhowmik, Sumonkanti Das

**Affiliations:** Department of Statistics, Shahjalal University of Science & Technology, Sylhet, 3114 Bangladesh

**Keywords:** Linear regression, Logistic regression, Height-for-age Z-score, Stunting

## Abstract

**Background:**

Logistic regression analysis is widely used to explore the determinants of child malnutrition status mainly for nominal response variable and non-linear relationship of interval-scale anthropometric measure with nominal-scale predictors. Multiple classification analysis relaxes the linearity assumption and additionally prioritizes the predictors. Main objective of the study is to show how does multiple classification analysis perform like linear and logistic regression analyses for exploring and ranking the determinants of child malnutrition.

**Methods:**

Anthropometric data of under-5 children are extracted from the 2011 Bangladesh Demographic and Health Survey. The analysis is carried out considering several socio-economic, demographic and environmental explanatory variables. The Height-for-age Z-score is used as the anthropometric measure from which malnutrition status (stunting: below −2.0 Z-score) is identified.

**Results:**

The fitted multiple classification analysis models show similar results as linear and logistic models. Children age, birth weight and birth interval; mother’s education and nutrition status; household economic status and family size; residential place and regional settings are observed as the significant predictors of both Height-for-age Z-score and stunting. Child, household, and mother level variables have been ranked as the first three significant groups of predictors by multiple classification analysis.

**Conclusions:**

Detecting and ranking the determinants of child malnutrition through Multiple classification analysis might help the policy makers in priority-based decision-making.

**Trial registration:**

“Retrospectively registered”

**Electronic supplementary material:**

The online version of this article (10.1186/s40795-017-0194-7) contains supplementary material, which is available to authorized users.

## Background

Globally 165 million under five children are stunted (short compared to their age), and about 1 million child deaths annually due to stunting [1]. In 2012, the World Health Organization (WHO) adopted an implementation plan for a global target of 40% reduction in stunting by 2025 [2]. The current child nutrition situation is very worse in developing countries [3], particularly in Southern Asia where stunting level is about 38% [4]. Bangladesh is one of the Southern Asian countries [5], where stunting level is above the WHO critical threshold (40%) in 39 out of 64 districts [6]. Recently Bangladesh has achieved lower stunting level (37% in 2013) compared to Pakistan (45% in 2012) and India (48% in 2006) [7], however, the rate is still high as per WHO threshold of high prevalence (30–39%) [3].

Government and policy makers routinely measure anthropometric indices like height-for-age (HAZ), weight-for-age (WAZ), and weight-for-height (WHZ) Z-scores for determining child malnutrition status. A child is called malnourished if any of his/her anthropometric indices is below −2.00 standard deviation (SD). This nutrition status variable is used to develop logistic model using nominal-scale explanatory variables to determine risk factors of child malnutrition in many previous studies [8–14]. A few studies have been found in literature where a linear regression model of an anthropometric index is developed using nominal-scale predictors. Since malnutrition status is determined from anthropometric measure, it can be examined whether the same predictors influence both nutrition index and malnutrition status.

In linear and logistic regression analyses, the explanatory variables are assumed to be linearly related to the interval-scale response variable and the logit respectively. Multiple classification analysis (MCA) relaxes this linearity assumption to fit an additive model for examining the significance of the predictors on both interval and nominal-scale response variables. The significant predictors cannot be ranked in linear/logistic model, however MCA can rank them based on their influence on the response variable [15]. The MCA also shows both bivariate and multivariate (absence and presence of other predictors respectively) relationships of a predictor with the response variable. The aim of this study is to show how the MCA provides similar results as linear and logistic regression analyses and additionally prioritizes the significant predictors. More specifically, the major goals of this article are (1) to determine risk factors of under-5 child malnutrition in Bangladesh considering both interval-scale HAZ and nominal-scale nutrition status as response variables, (2) to compare linear and logistic regression approaches to MCA approach empirically, and (3) to show how the MCA provides additional information over linear and logistic regression analyses.

## Methods

### Study materials

This study uses children anthropometric data collected in the nationwide 2011 Bangladesh Demographic and Health Survey (BDHS) [16]. The country was stratified into 20 strata according to rural and urban enumeration areas of 7 divisions. A nationally representative sample is drawn following a two-stage stratified sampling design: 600 clusters (393 from rural and 207 from urban areas) are drawn at the first stage and then 30 HHs were systematically selected from the selected enumeration areas which are called clusters in the BDHS survey. A total of 17,141 HHs from where 17,842 ever married woman were selected to collect socio-economic, demographic, environmental, and health care related information. Anthropometric measures age, height, and weight are collected for the children aged under 5 years. In total about 8281 children under age five at the interview date were selected for measuring height and weight, however measurements were completely collected from 7826 children (few were absent or refused to provide height and weight), of which 7647 children had plausible anthropometric information for calculating anthropometric measures. As the 2011 BDHS data, the same children data has been utilized in this study. The characteristics of the study population are detailed in the 2011 BDHS report [16].

### Child nutritional status

In 2011 BDHS data, three anthropometric indices HAZ, WAZ, and WHZ are calculated based on WHO 2006 Child Growth Standards [17]. These anthropometric measures are routinely analyzed to provide assessment of child nutritional status [18]. The HAZ represents the chronic nutrition measure of under five children. A child is defined as stunted when his/her HAZ is less than −2.00 SD. Let *y*_*i*_ denotes the HAZ of a child and *z*_*i*_ indicates the nutrition status of the child: nourish (*z*_*i*_ = 0 when y_*i*_ ≥  − 2.0) or stunted (*z*_*i*_ = 1 when *y*_*i*_ <  − 2.0). Also, let the vector **x**_*i*_ denotes the values of the explanatory variables which are assumed linearly related to the interval-scale response variable *Y*and to a logit link function of the probability of being malnourished. Under this consideration, the development of the linear model (LM) and interval-scale MCA (IS-MCA) model for Y, and binary logistic model (BLogM) and nominal-scale MCA (NS-MCA) model for Z are briefly discussed in the following sub-sections.

### Linear regression analysis

A linear regression model (LM) is fitted using either least squares or maximum likelihood (ML) method considering the underlying assumptions including linearity of explanatory variables with response variable. Goodness of a fitted linear model is assessed mainly by F-test for overall model and R-squared measures. The performance of the fitted model can also be measured by comparing the observed nutrition status (based on observed HAZ) with the predicted nutrition status (based on predicted HAZ). The proportion of children classified correctly in such way is termed as correct classification rate in this paper.

### Logistic regression analysis

Logistic regression model predicts the probability of a child being malnourished instead of predicting his/her nutrition measure given the values of explanatory variables. A binary logistic model (BLogM) can be written as ${\mathrm{log}}_e\left[{\pi}_i/\left(1-{\pi}_i\right)\right]={\mathbf{x}}_i^T\boldsymbol{\upbeta}$ where ${\pi}_i={p}_r\left({z}_i=1|{\mathbf{x}}_i^T\right)$ $={e}^{{\mathbf{x}}_i^T\boldsymbol{\upbeta}}/\left(1+{e}^{{\mathbf{x}}_i^T\boldsymbol{\upbeta}}\right)$ is the conditional probability of *z*_*i*_ = 1 given ${\mathbf{x}}_i^T$. This BLogM is fitted using ML method with a suitable iterative process such as Newton’s method [19]. The goodness of a fitted BLogM is assessed mainly by an R-squared statistic and a goodness-of-fit statistic. The mostly used R-squared statistics available in common statistical software are McFadden [20] and Cox and Snell R-squared statistics [21] (Nagelkerke’s pseudo R-squared statistics [22] in SPSS). The Hosmer-Lemeshow (H-L) test, the most commonly used goodness-of-fit test for BLogM, assures linearity between the log-odds and the explanatory variables [23]. An alternative measure of goodness-of-fit is to compare the observed nutrition status to the predicted nutrition status based on the fitted BLogM, which helps to find out false negative and false positive classification rates [23]. In SPSS, an overall classification rate is reported based on a cut-off point of $\widehat{p}=0.50$. Overall performance of the BLogM model can be assessed by comparing classification rates obtained from full and null models. This assessment is relevant to the area under receiver operating characteristic curve of a fitted model [24]. Significance of a predictor can be assessed by likelihood ratio, Wald, and score tests [19], which are asymptotically equivalent [25].

### Multiple classification analysis

The linear relationship between response and explanatory variables is in question when the explanatory variables are nominal in nature. The MCA is a multivariate technique which relaxes this linearity assumption [15] and assess the interrelationship through an additive model. The MCA determines the effect of each predictor on the response before and after adjustments for its inter-correlations with other predictors. Each category of a nominal explanatory variable (factor) is considered as an independent predictor (dummy variable), and uncorrelated to other explanatory variables. The advantage of MCA over multiple linear regression analysis is that it can handle any form of interrelationships between the explanatory and the response variables. Also, the similar additive model can be developed for either interval or nominal-scale response variable with the same explanatory variables.

In MCA model, a coefficient is assigned to each category of each explanatory variable in such way that the response value for an individual is the sum of the coefficients assigned to all categories that represent the individual characteristics, grand mean of the response and a random error term. Thus MCA models for *Y* and nominal-scale nutrition status *Z* can be expressed by the same model as *ν*_*j* … *n*_ = *μ* + *a*_*j*_ + *b*_*k*_ +  .  …  + *e*_*j* … *n*_, where *ν*_*j* … *n*_ is the response value for a child who falls into *j* ^*th*^ category of predictor *A*, *k* ^*th*^ category of predictor *B* and so on; *μ* is the grand mean, *a*_*j*_ is the added effect of *j* ^*th*^ category of predictor *A* (difference between *μ* and mean response value of *j* ^*th*^category of *A*), *b*_*k*_is the added effect of *k* ^*th*^category of predictor *B* (difference between *μ* and mean response value of *k* ^*th*^ category of *B*); *e*_*j* … *n*_is the error. The coefficients are estimated via a technique like iterative least squares method. The diagnostic of the fitted model can be done by checking whether all the predictors can explain a significant proportion of variation. For details please see Andrews et al. [15] and Nagpaul [26].

For assessing the importance of a factor (the degree of relationship), two correlation ratios called eta (*η*_*i*_) and beta (*β*_*i*_) statistics are calculated from the model before and after the adjustment of other predictors respectively [27]. Eta and beta values indicate the proportion of variation in the response variable accounted by each predictor. The beta value indicates the importance of a predictor on the response variable based on which the predictors can be ranked [28]. The comparison between eta and beta values helps one to examine whether the importance of a predictor in a bivariate situation remains in a multivariate design. The specific forms of eta and beta for a predictor are illustrated by Nagpaul [26]. The gain of MCA over regression analysis is estimating the effect of each predictor on the response variable with or without considering the effects of all other predictors without any constraints [26].

### Explanatory variables

A number of explanatory variables at children level (age, birth weight, and birth interval), mother’s level (education and nutrition status), household level (wealth status and family size), community level (rural and urban areas), and regional level (division) are considered in the study for developing all the LM, BLogM, IS-MCA, and NS-MCA models. The household and community information were collected from the household head, while children and mother’s information were collected from the mothers. The considered explanatory variables are identified as significant predictors of child malnutrition in many child malnutrition researches [8, 29–35] by developing BLogM models considering Z as response variable. In this study, linear and IS-MCA models are developed to see how the considered explanatory variables influence the interval-scale HAZ score. The considered LM, BLogM, IS-MCA and NS-MCA models are developed using the LM, LOGIT and ANOVA with MCA functions of SPSS (22.0 version) respectively.

## Results

The developed LM, BLogM, IS-MCA and NS-MCA models shown in Table 1 and Table 2 indicate that all the predictors included in the models are significant as expected from previous studies.

**Table 1:** Estimated regression coefficients of linear regression model (LM) for Height-for-Age Z-score (HAZ), and adjusted mean predicted (APM) HAZ calculated from interval-scale multiple classification analysis (IS-MCA) model by different socio-demographic factors, BDHS 2011

Background Characteristics	No. of children	Estimated regression coefficient of LM	Mean predicted HAZ from IS-MCA
		Β	APM
*Child*’*s age in months (Reference Category: <12)*			
<12	1484		−0.9442
12–23	1443	−0.938***	−1.8826
24–35	1441	−0.940***	−1.8843
36–47	1679	−0.903***	−1.8470
48–59	1600	−0.808***	−1.7527
*Child*’*s birth weight (Reference Category: Large)*			
Large	1086		−1.4872
Average	5262	−0.147**	−1.6345
Small	1299	−0.455***	−1.9421
*Birth interval in months (Reference Category: 48+)*			
<24	587	−0.268***	−1.8869
24–47	1879	−0.108**	−1.7265
48+	5181		−1.6187
*Mother*’*s education (Reference Category: Higher)*			
Illiterate	1449	−0.484***	−1.7726
Primary	2330	−0.462***	−1.7503
Secondary	3260	−0.340***	−1.6284
Higher	608		−1.2881
*Mother*’*s BMI(kg/m2) (Reference Category: 18.5–24.99)*			
<18.5	2116	−0.142***	−1.7922
18.5–24.99	4576		−1.6504
25+	955	0.191***	−1.4597
*Household economic status (Reference Category: Richest)*			
Poorest	1682		−2.0438
Poorer	1489	−0.838***	−1.7987
Middle	1456	−0.593***	−1.6967
Richer	1493	−0.491***	−1.5476
Richest	1527	−0.342***	−1.2060
*Family size (number of members) (Reference Category: Middle)*			
Small (<4)	761	−0.086	−1.7057
Middle (4-6)	4248		−1.6192
Large (7+)	2638	−0.110**	−1.7294
*Place of residence (Reference Category: Urban)*			
Urban	2342		−1.7196
Rural	5305	0.077**	−1.6421
*Region (Division) (Reference Category: Barisal)*			
Barisal	837		−1.6966
Chittagong	1516	0.012	−1.6844
Dhaka	1272	0.003	−1.6932
Khulna	894	0.109	−1.5878
Rajshahi		0.261***	−1.4357
Rangpur	916	0.003	−1.6937
Sylhet	993	−0.104	−1.8004
Goodness-of-fit		*F*_26,7620_ = 59.952; *p*-value < 0.001	*F*_26,7620_ = 59.952; *p*-value < 0.001
R^2^ value		0.170	0.170

*** *P* < 0.001; ** *P* < 0.01; * <0.05

**Table 2:** Estimated regression coefficients of binary logistic regression model (BlogM) for child malnutrition status defined as height-for-age Z-score less than −2.00, the corresponding odds ratios (ORs), and adjusted predicted proportion (APP) of malnourished children from nominal-scale multiple classification analysis (NS-MCA) model, BDHS 2011

Background Characteristics	Estimated regression coefficient of BlogM and OR		Predicted proportion from NS-MCA
	Β	OR	APP
*Child*’*s age in months (Reference Category: <12)*			
<12			20.83
12–23	1.406	4.081***	48.89
24–35	1.314	3.722***	46.78
36–47	1.284	3.610***	46.13
48–59	1.027	2.792***	40.42
*Child*’*s birth weight (Reference Category: Large)*			
Large			33.61
Average	0.318	1.374**	39.95
Small	0.753	2.123***	49.47
*Birth interval in months (Reference Category: 48+)*			
<24	0.383	1.467***	47.35
24–47	0.208	1.231**	43.47
48+			38.90
*Mother*’*s education (Reference Category: Higher)*			
Illiterate	0.642	1.900***	44.23
Primary	0.592	1.807***	42.92
Secondary	0.418	1.519***	39.08
Higher			32.10
*Mother*’*s BMI(kg/m2) (Reference Category: 18.5–24.99)*			
<18.5	0.270	1.310***	45.91
18.5–24.99			39.81
25+	−0.346	0.708***	33.19
*Household economic status (Reference Category: Richest)*			
Poorest	1.109	3.031***	51.26
Poorer	0.869	2.385***	45.68
Middle	0.673	1.961***	41.20
Richer	0.451	1.570***	36.48
Richest			27.71
*Family size (number of members) (Reference Category: Middle)*			
Small (<4)	0.173	1.189*	42.58
Middle (4–6)			39.00
Large (7+)	0.177	1.194**	42.80
*Place of residence (Reference Category: Urban)*			
Urban			42.84
Rural	−0.153	0.858*	39.71
*Region (Division) (Reference Category: Barisal)*			
Barisal			41.29
Chittagong	0.011	1.011	41.54
Dhaka	0.108	1.114	43.65
Khulna	−0.198	0.820	37.34
Rajshahi	−0.400	0.671***	33.12
Rangpur	−0.057	0.944	40.12
Sylhet	0.158	1.171	44.60
Goodness-of-fit	H-L test: ${\chi}_{(8)}^2$=10.090; p-value: 0.259		*F*_*26,7620*_ = 41.329; p-value < 0.001
	Omnibus test: ${\chi}_{(26)}^2$=1025.05; p-value < 0.001		
R^2^ value	0.125		0.124

*** *P* < 0.001; ** *P* < 0.01; * <0.05

### Significance and association of the explanatory variables with child malnutrition

The fitted LM shows that the mean HAZs for the older-groups children are about 0.90 Z-score lower than the mean HAZ of infants. The IS-MCA model also shows that the adjusted predicted mean (APM) of HAZ are about −0.94 Z-score for infants and more than −1.80 Z-score for older children (Table 1). The fitted BLogM shows that children aged 12–23 months have the higher risk of being malnourished compared to infants and then the risk decreases gradually with the age categories (Table 2). The NS-MCA model confirms the results of BLogM: the lowest adjusted predicted proportion (APP) of stunted children in the infant group (20.8%) and highest proportion in the 12–23 group (48.9%). In case of child birth weight, the fitted models show that the small birth weight children have about 0.46 SD lower HAZ, about 16% higher APP and more than double risk of being stunted (APM: -1.94, APP: 49.5 & OR: 2.1) compared to the children with large birth weight (APM: -1.49, APP: 33.6 & OR: 1). For birth interval, the coefficients in LM and the APMs in IS-MCA model decrease significantly with the decrease of birth interval category. The MCA models also show that the children with less than 2-years birth interval have higher stunting (APP: 47%) and lower mean HAZ (APM: -1.89 SD). It is noted that here the reference group represents children with first birth order or 48+ month previous birth interval.

The fitted LM and IS-MCA show increasing regression coefficients and APMs respectively (Table 1), while BLogM and NS-MCA show decreasing odds ratios and APPs respectively with the improvement of mother’s education (Table 2). The models show that the illiterate mothers’ children have about 0.50 lower HAZ and 1.9 times higher risk of being stunted compared to those of higher educated mothers (APMs: −1.77 and −1.29 SD; APPs: 44.0% and 32% for illiterate and higher). In case of mothers’ body mass index (BMI), Table 2 shows that mothers with lower BMI have higher risk of having stunted children (OR: 1.31 and APP: 46.0%) compared to mothers with higher BMI (OR: 0.79 and APP: 33%).

The estimated regression coefficients and mean HAZs are found to increase with the order of household economic status (Table 1). The results of BLogM and NS-MCA models (Table 2) indicate that the children living in poorest households are more likely to be stunted (OR = 3.0 and APP: 51%) than those of richest households (OR = 1.0 and APP: 28%). For family size, BLogM and NS-MAC models show slightly higher odds ratios (OR: 1.19 and 1.19) and APPs (43% and 43%) for the small and large families.

The fitted models indicate that the children living in rural areas have slightly lower mean HAZ and risk of being stunted (APM: -1.64, APP: 39.71) than those living in urban area (APM: -1.72, APP: 42.84). However, the unadjusted predicted mean HAZ (Urban: −1.46 SD and Rural: −1.76 SD) and unadjusted predicted proportion of stunted children (Urban: 35.23% and Rural: 43.07%) are found lower in urban areas in the IS-MCA and NS-MCA models respectively. The analysis by regional settings “Division” indicates that the children of *Rajshahi* division had significantly lower risks of being malnourished (OR: 0.67 & APM: -1.44) compared to the other divisions, while children of *Sylhet* division are more vulnerable (OR: 1.17, APM: -1.80).

### Ranking of the risk factors

Both the MCA models of HAZ and nutrition status shown in Table 3 indicate that the highest variation in the response is contributed by the children’s age-category variable (*β*_*i*_=0.254 & 0.206 respectively) followed by the household socio-economic status (*β*_*i*_=0.201 & 0.166) and children’s birth weight (*β*_*i*_=0.096 & 0.092). The place of residence is ranked at the last position due to its lowest contribution in both cases (*β*_*i*_=0.025 & 0.029) given other explanatory variables. Most of the predictors except mothers’ education status and current nutrition status possessed the same ranks in both the models. Mother’s Education status is ranked as fourth and nutrition status as sixth contributors in IS-MCA model, and they reverse the position in NS-MCA model.

**Table 3:** Priority index and significance for the risk factors of child malnutrition in Bangladesh via interval-scale (IS-MCA) and nominal-scale (NS-MCA) multiple classification analysis models

Risk factors	Priority index from IS-MCA model					Priority index form NS-MCA model			
	*df*	*η*_*i*_	*β*_*i*_	F	*p*-value	*η*_*i*_	*β*_*i*_	F	*p*-value
Child’s age in months	4	0.254	0.254	146.165	0.000	0.207	0.206	91.542	0.000
Child’s birth weight	2	0.111	0.096	41.555	0.000	0.107	0.092	36.567	0.000
Birth interval in months	2	0.117	0.056	13.206	0.000	0.108	0.056	12.277	0.000
Mother’s education	3	0.225	0.091	18.570	0.000	0.186	0.067	9.223	0.000
Mother’s BMI (kg/m2)	2	0.165	0.071	19.613	0.000	0.157	0.079	23.309	0.000
Wealth index	4	0.263	0.201	50.111	0.000	0.217	0.166	32.104	0.000
Family size (number of members)	2	0.013	0.037	6.082	0.002	0.009	0.038	5.996	0.002
Region(Division)	6	0.105	0.072	7.630	0.000	0.098	0.071	7.022	0.000
Place of resident	1	0.098	0.025	4.471	0.035	0.074	0.029	5.681	0.017

Note: *η*_*i*_ and *β*_*i*_ indicates priority indices before and after the adjustment of other predictors respectively

### Goodness of the fitted models

The ANOVA tests for LM and IS-MCA models (*F*_26,7620_ = 59.952, *p*-value <0.001) confirm the goodness of the models. For logistic model, the omnibus test (${\chi}_{(26)}^2$=1025.05, p-value <0.001) confirms the overall significance of regression coefficients and the H-L test (${\chi}_{(8)}^2$=10.09, p-value = 0.259) confirms the linearity of logit with the explanatory variables. Adequacy of the NS-MCA model is also confirmed via ANOVA test (*F*_26,7620_ = 41.329, p-value <0.001). The predictive powers of the fitted MCA models are about 17% and 13% when the response variables are considered as interval and nominal-scale respectively. Effects of the explanatory variables in both the MCA models shown in Table 3 are found statistically significant. The classification rates shown in Table 4 suggest that nutrition status of about two-third children are correctly identified by the LM (65.3%) and BLogM (66.4%) models.

**Table 4:** Correct classification rate of children nutrition status based on height-for-age Z-score (HAZ) as either malnourish (HAZ < −2.0) or nourish (HAZ ≥ −2.0) from linear regression (LM) and logistic regression (BLogM) models, and the overall correct classification rate of children nutrition status by LM and BLogM models, BDHS 2011

Observed nutrition status	Predicted nutrition status by LM		Correct classification by LM (%)	Predicted nutrition status by BLogM		Correct classification by BLogM (%)
	Nourish	Malnourish		Nourish	Malnourish	
Nourish	3609	913	79.8	3541	996	78.0
Malnourish	1657	1432	46.4	1571	1539	49.5
Overall correct classification rate (%)			65.3			66.4

Note: Correct classification rates are row wise percentages

## Discussion

The study has attempted to identify the risk factors of child malnutrition considering both interval-scale nutrition measure and nominal-scale nutrition status, and then rank the risk factors based on a priority index via MCA. The findings of the study clearly indicate that the MCA models provide results comparable to those of linear and logistic regression analyses. In case of exploring significant predictors, all the fitted models behave in the same line and show that all the assumed predictors are significant. The fitted models provide similar information in different ways such as: linear and logistic models provide respectively the mean change in HAZ and the risk of being malnourished for a specific group compared to a reference group, while the similar interpretation can be made from MCA by comparing the APM of HAZ and APP of stunted children for a compared group with those of the reference group. The ORs from BLogM and the APPs from NS-MCA model are positively related as the higher the OR of a group, the higher the corresponding APP. It can be said that the APMs can be calculated from the fitted LM and an approximate ORs can be calculated from APPs of NS-MCA model.

The analysis of this article supports the previous findings [8, 10, 29, 31] that Bangladeshi children aged under 5 years are in a good nutrition status in their first year of life, and then the nutrition status becomes worse with the age of children. The alarming issue is that the proportion of malnourished children for all the older age-group are more than the cut-off point of “high prevalence” stunting (30–39%).

Children who were born with lower weight are found to have higher risk of being malnourished in future as in some recent studies [35–37]. Similarly, children birth interval is found inversely related with their nutrition status as expected [38, 39]. The study confirms that the higher the birth interval the higher the mean HAZ score and the lower risk of being stunted.

Mother’s education is an important hidden factor of children health and nutrition status. Like previous researches [10, 31, 40–42], we also observed that mother’s education status is positively related with HAZ and negatively related with the risk of having stunted children. Mothers’ current maternal nutrition, measured via BMI, is also found positively related with child nutrition status as previous studies of Bangladeshi children [10, 31]. This study also found that mothers with adequate nutrition status are less likely to have stunted children.

Household factors are strong predictors of children nutritional status [42, 43]. Usually, children in households having higher income have better nutritional status than that of lower income households. The study also found that the children of wealthy households have better HAZ score and lower risk of being stunted. The disaggregation by wealth status reveals that more than half of the children belonging to poorest households are malnourished while the proportion is below 30% for the richest households. Household size has also impact on the child nutrition status [31, 42]. Children born in small (3 or less) and large (7 or more) families are more likely to be malnourished in comparison to those born in medium-size households.

A significant residential difference in stunting level is observed in Bangladesh like other studies of child malnutrition [12, 14, 42, 44]. However, it is observed that the children of rural areas have slightly higher risk of being malnourished compared to the urban children when the influence of other variables are considered. These converse results by rural-urban residence might be due to the influence of others predictors. One of such influential facts may be the inter-relationship between mother’s education and household wealth status shown in Additional file 1 Table S1. The table shows that the richer households in rural area have less illiterate and primary educated mothers compared to those in urban areas. Another reason behind this fact may be considering more slums in urban areas specially in capital city Dhaka where bulk share of population is living.

The location variable “division” is also found significantly associated with child chronic malnutrition in all models as previous nutrition studies of Bangladeshi children [8, 10, 31, 35]. The “division” variable is found to have significant contribution to all LM, BLogM, IS-MCA and NS-MCA models, though the variation in mean HAZ and proportion of stunted children by regional setting is varying particularly due to poor condition in Sylhet division and better condition in Khulna division. The main concern for the government is that only in Khulna, the adjusted proportion of stunted children is found close the MDG target of 33%.

In most of the studies the risk factors are only identified by fitting BLogM model but not order them according to their contribution. The study shows that MCA provides a way to rank the significant variables as per their relative importance on the response variable. The findings from MCA suggest that the child-level predictors are the main contributors for explaining the variation of the child nutrition status. Household level and mother level predictors have the second and third most contributions. The variations in child nutrition status due to regional settings and residential place are found very negligible and are ranked as the lower contributors.

## Conclusions

Though much improvement has been done in reduction of national level child malnutrition in Bangladesh, disaggregate level malnutrition is still very high. Multiple classification analyses show that more than two-fifths children of different demographic (particularly children aged 12–23 months, with lower birth weight and birth interval) and socio-economic (particularly illiterate and malnourish mothers, and poor households) groups are chronically malnourished. For achieving the WHO global target on stunting in Bangladesh by 2025, it is necessary to reduce these disaggregate level malnutrition. To take proper initiatives for reducing the disaggregated level malnutrition, the significant predictors are usually identified by utilizing the logistic regression analysis. In this study, MCA has been implemented along with the logistic regression analysis considering both nominal and interval-scale nutrition variables. Both types of analyses identify the same predictors attributed to the poor nutrition status of children. The models LM and BLogM help to find the influential predictors only, while the MCA provides the same information with an extra facility of ranking those significant predictors. Ranking of the predictors might help to the policy makers for lining up their interventions. In addition, the results of MCA will help to a non-statistician for understanding how much more or less the predicted mean Z-score or stunting level for a target group compared to the reference group. The application of MCA approach and its findings in this study also suggest that MCA (with/out LM or BLogM) can be used in other public health studies like episode of diarrhoeal diseases or acute respiratory infection with the aim of exploring and ranking the risk factors.

## Additional file

Additional file 1: Table S1. Description of data: Distribution of mothers by household (HH) wealth status, mother’s highest level education status and residential place, BDHS 2011. (PDF 185 kb)

## Abbreviations

ANOVA: Analysis of variance
APM: Adjusted predicted mean
APP: Adjusted predicted proportion
BDHS: Bangladesh Demographic and Health Survey
BLogM: Binary logistic regression model
BMI: Body mass index
HAZ: Height-for-age Z-score
HH: Household
H-L: Hosmer-Lemeshow
IS-MCA: Interval-scale MCA
LM: Linear model
MCA: Multiple classification analysis
ML: Maximum likelihood
NIPORT: National Institute of Population Research and Training
NS-MCA: Nominal-scale MCA
OR: Odds ratio
WAZ: Weight-for-age Z-score
WHO: World Health Organization
WHZ: Weight-for-height Z-score
