# Supplementary material for: On exploring and ranking risk factors of child malnutrition in Bangladesh using multiple classification analysis
Source: BMC Nutr. 2017 Sep 7;3:73. doi: 10.1186/s40795-017-0194-7 (PMC7050713; doi:10.1186/s40795-017-0194-7)
Supplement: Supplementary file 1 — Description of data: Distribution of mothers by household (HH) wealth status, mother’s highest level education status and residential place, BDHS 2011. (PDF 185 kb) [file 40795_2017_194_MOESM1_ESM.pdf]

Appendix Table 1

Distribution of mothers by household (HH) wealth status, mother's highest level education status and residential place, BDHS 2011

Residential Place	HH Wealth Status	Mother's Education Status				Total
		Illiterate	Primary	Secondary	Higher	
Urban	Poorest	30.3	47.1	21.6	1.0	208
	Poorer	22.3	43.1	33.5	1.1	188
	Middle	13.7	38.8	43.8	3.7	299
	Richer	15.7	31.4	45.8	7.1	605
	Richest	4.9	14.0	51.2	29.8	1042
Rural	Poorest	43.6	38.9	17.2	0.2	1474
	Poorer	23.4	38.4	37.5	0.8	1301
	Middle	11.8	32.1	51.5	4.6	1157
	Richer	6.6	22.9	60.5	10.0	888
	Richest	2.9	10.7	69.1	17.3	485
	**Total**	18.9	30.5	42.6	8.0	7647

Note: Percentages are row wise
